# Editorial and Miscellaneous

**Published:** 1863-11

**Authors:** 


### EDITORIAL AND MISCELLANEOUS.

*Clinical Lectures on Diseases of the Eye and Ear.*—With this month we commence the publication of a series of clinical lectures on Diseases of the Eye and Ear by E. L. Holmes, M. D., Surgeon to the Chicago Eye and Ear Infirmary, and Lecturer on the same subjects in Rush Medical College. Our readers who have been familiar with the occasional essays of Dr. Holmes, as they have appeared in this and other medical journals, will, we are sure, join with us in felicitation that he has consented to place the results of his large attainments, extensive practice and close scientific observation, in this permanent form.

We are not lovers of specialties as such, but when we find the cultivation of specialties based upon thorough general professional acquirement, the case is wholly changed. When our own eyes and ears become afflicted with any of the ills to which those organs are heirs, we shall ask no better adviser than Dr. Holmes. Unassuming and retiring in his disposition, it has been a work of no common difficulty for us to induce him from time to time to come before the professional public, and now we write this little notice of his professional merits, with serious apprehensions of his high displeasure.

---

*Rush Medical College.*—It will probably gratify the alumni and other friends of Rush Medical College to hear that the class in attendance the present session is far larger than any other previously assembled. It has been found necessary to considerably increase the number of seats and the demand is still for more, and this demand is being promptly met by constant additions. It is now confidently expected that at the termination of this, its twenty-first year, the College will put on a "freedom suit" in the shape of buildings capable of accommodating double the present number of students, with a commodious and spacious hospital attached, under the full and immediate control of the institution. A college hospital has become necessary, not only for the interests of the College, but for those of the city and county. The City Hospital is disadvantageously located, and during the war has been "confiscated" to the sole uses of the U. S. Government. Mercy Hospital, so called, is even more remote, and unequal, in every point of view, either to the exigencies of our city or to the convenience of clinical teaching. Practically, it is of no service save to a very limited number of students. It seems to be admitted generally that it has room enough for those at present in attendance, although it would be a mere bagatelle for old Rush. Very superior facilities are now afforded for clinical teaching by the Marine Hospital, which a very large number of the class are taking advantage of. Meanwhile the College Cliniques furnish an amount and variety of illustration fully up to the wants and time of the class, and unsurpassed west of New York and Philadelphia.

All gentlemen of the profession, both in the city and the country, are invited to visit the College whenever so disposed, and they are hereby assured that there is no Register of visitors kept in the ante-room, the contents of which will be published in the next annual catalogue.

---

*Pyrophosphate of Iron and Calisaya Bark.*—The attention of readers is directed to the change in the advertisement of this very elegant and really serviceable chalybeate tonic as prepared by Mr. Sargent. In reply to numerous inquiries he publishes the proportions of the active ingredients. Our friends may rely implicitly upon the statements of Mr. Sargent with regard to this or any other medicine he prepares, as we know him to be an accomplished pharmaceutist, and a gentleman of the highest integrity. Mr. S., and several other gentlemen we could name, are rapidly bringing the apothecary *business* to the level of a learned profession and a fine art.

---

The distinction between symptoms and signs, so often confounded, is well illustrated by the following from Captain F. B. Head's Rough Notes, taken during some rapid journeys across the Pampas, p. 257:

"The Gaucho pointed to the sky, and said, 'See! there is a lion.' I started from my reverie, and strained my eyes, but to no purpose; and he showed me at last, very high in the air, a number of large vultures, which were hovering without moving; and he told me they were there because there was a lion devouring some carcase, and that he had driven them away from it. We shortly afterwards came to a place where there was a little blood in the road, and for a moment we stopped our horses to look at it. I observed, perhaps some person had been murdered there; the Gaucho said, 'No;' and, pointing to some foot-marks which were near the blood, he told me that some man had fallen, that he had broken his bridle, and that while he was standing to mend it, the blood had evidently come from his horse's mouth. I observed, perhaps it was the *man* who was hurt; upon which the Gaucho said, 'No;' and, pointing to some marks a few yards before him on the path, he said, 'for, see, the horse set off at a gallop.'"

The Englishman might have speculated long and wisely upon the flight of vultures, upon the structure and functions of their organs, even to the minutiæ of a feather, and yet never been so far benefitted by his speculations as to diagnosticate a lion. What are mere symptoms to one, become through a knowledge of their relationship, valuable signs to another.—*Wyman.*

---

NOTICE TO THE MEDICAL PROFESSION.

Having been engaged in the practice of my Profession at this place for eight years, and, wishing to retire, I offer for sale (*at a sacrifice*) my splendid Residence, Office, and everything belonging to a HOME, and the best location for a Country and small Village practice in the State of Iowa. Amount of practice ranging from $2000 to $4000—just as a man can stand it and is disposed to make it. Terms, $3000. $1500 cash down; balance in three equal annual installments, (secured on the property,) bearing interest.

*I will sell.* For further particulars address,

DR. KITTLE,

(Box No. 2.) MARSHALL, Henry Co., IOWA.

